# Frailty, All-Cause Mortality, and Hospitalization in Patients on Maintenance Hemodialysis: A Systematic Review and Meta-Analysis

**DOI:** 10.3390/jcm14144914

**Published:** 2025-07-10

**Authors:** Maurizio Bossola, Ilaria Mariani, Manuela Antocicco, Gilda Pepe, Enrico Di Stasio

**Affiliations:** 1 Servizio Emodialisi, Dipartimento di Scienze Mediche e Chirurgiche, Università Cattolica del Sacro Cuore, Largo A.Gemmelli 8, 00168 Rome, Italy; ilaria.mariani04@icatt.it (I.M.);; 2 Policlinico Universitario Fondazione Agostino Gemelli IRCCS, 00168 Rome, Italy; 3 Dipartimento Scienze dell’invecchiamento, Neurologiche, Ortopediche e Della Testa-Collo, 00168 Rome, Italy; 4 Dipartimento di Scienze Mediche e Chirurgiche, Università Cattolica del Sacro Cuore, 00168 Rome, Italy; 5 Dipartimento di Scienze Biotecnologiche di Base, Cliniche Intensivologiche e Perioperatorie, Università Cattolica del Sacro Cuore, 00168 Rome, Italy

**Keywords:** hemodialysis, frailty, mortality, hospitalization

## Abstract

**Background/Objective**: In recent years, three systematic reviews examining the relationship between frailty and mortality in chronic hemodialysis patients have been published; these reviews employed different inclusion criteria and methodologies, leading to conflicting results. The present study aimed to determine whether frailty is associated with an increased risk of all-cause mortality and hospitalization in patients on maintenance hemodialysis. **Methods**: The research was conducted in April 2024 using the following databases: MEDLINE via PubMed (1985 to present) and Web of Science Core Collection via Clarivate (1985 to present), with a combination of keywords to capture hemodialysis, frailty, and mortality. **Results**: We included 23 studies in the analysis, with a total of 10,333 patients (5592 frail and 4741 non-frail). The number of patients in each individual study ranged from 93 to 1652. Adjusted mortality data that accounted for patient characteristics and treatment variables was available from six studies (1034 patients) with a follow-up period of 12 months and revealed an increased all-cause mortality risk in frail patients in the random effects model (pooled OR, 3.28; 95% CI, 1.72–6.29). Moderate heterogeneity was observed in this analysis (Chi^2^ = 14.06, df = 5, (*p* = 0.02); I^2^ = 64%). Adjusted mortality data that accounted for patient characteristics and treatment variables was available from 21 studies (8757 patients) with any follow-up period and revealed an increased all-cause mortality risk in frail patients in the random effects model (pooled adjusted OR 2.47; 95% CI, 1.85–3.29). Moderate heterogeneity was observed in this analysis (Chi^2^ = 55.1, df = 20, (*p* < 0.0001); I^2^ = 64%). All-cause hospitalization data was available from 15 studies (6349 patients) with any follow-up period and revealed an increased all-cause hospitalization risk in frail patients in the random effects model (pooled adjusted OR 2.19; 95% CI, 1.72–2.78). Moderate heterogeneity was observed in this analysis (Chi^2^ = 40.9, df = 13; *p* < 0.0001; I^2^ = 68%). No obvious asymmetry, indicating no clear evidence of publication bias, was observed. **Conclusions**: Frailty is a significant risk factor for all-cause mortality and all-cause hospitalization in patients on maintenance hemodialysis and presents clinicians with important challenges in routine clinical practice.

## 1. Introduction

Frailty is an age-related clinical condition characterized by a state of increased vulnerability, resulting from a decline in reserve and function across multiple organ systems, and a reduced ability to cope with stressors [1,2]. When stressor events such as acute illness occur, the functional capacity of a frail person rapidly deteriorates. Frailty is commonly associated with an increased risk of mortality, morbidity, hospitalizations, falls, admission to long-term care, disability, fractures, worsening mobility, loneliness, lower quality of life, depression, cognitive decline, and dementia [1,2].

Fried et al. defined frailty as a condition meeting three out of the five phenotypic criteria indicating compromised energy, such as low grip strength, low energy, slowed walking speed, low physical activity, and/or unintentional weight loss [3].

The prevalence of frailty varies greatly across studies and among low-income, middle-income, and high-income countries. It has been reported recently that, in high-income countries, the weighted average estimated prevalence of frailty is 11% [1,2]. However, the prevalence of frailty is significantly higher in patients affected by chronic diseases. It has been reported that frailty is present in 50–55% of long-term care residents, in 10–30% of HIV patients, and in about 45% of cancer patients [1,2].

Frailty is very common in end-stage renal disease patients receiving maintenance hemodialysis with a prevalence ranging from 6.0 to 82.0% and a pooled prevalence of 34.3% (95% confidence interval (CI) 24.5–44.1%; z = 6.87; *p* = 0.00) [4]. Differences in the prevalence of frailty in hemodialysis patients across studies may be due to numerous reasons, such as differences in age, malnutrition, morbidity, and the inclusion of prevalent or incident patients. In addition, differences in the methods used to detect frailty may be present.

In recent years, three systematic reviews and meta-analyses have been published with different inclusion criteria and methodologies, leading to conflicting results [5,6,7]. The review by Lee et al. included only seven studies [5]. The review by He et al. included both patients receiving peritoneal dialysis and hemodialysis [7]. Since then, numerous other studies have investigated the relationship between frailty and mortality and between frailty and hospitalization in patients on maintenance hemodialysis, but the results remain uncertain [8,9,10,11,12,13,14,15,16,17,18,19,20,21,22,23,24,25,26,27,28,29]. Therefore, it is of paramount importance to assess the risk of mortality and hospitalization associated with frailty in patients on maintenance hemodialysis. The present systematic review aims to determine whether frailty is associated with an increased risk of mortality and hospitalization in end-stage renal disease (ESRD) patients on maintenance hemodialysis.

## 2. Methods

The present analysis was conducted in accordance with PRISMA (Preferred Reporting Items for Systematic Reviews and Meta-Analyses) guidelines and was prospectively registered in the International Prospective Register of Systematic Reviews in Health and Social Care (PROSPERO, ID number CRD42024535871) 26 April 2024.

### 2.1. Eligibility Criteria

Studies were eligible for inclusion if they were published in a peer-reviewed journal and met the following inclusion criteria: (1) cohort studies, prospective or retrospective, on adult patients (over 18 years of age); (2) patients with end-stage renal disease on maintenance hemodialysis; (3) compared all-cause mortality in patients with and without frailty assessed with any tool; (4) compared all-cause hospitalization in patients with and without frailty assessed with any tool; (5) all-cause mortality and all-cause hospitalization expressed as a hazard ratio, risk ratio, or odds ratio. We excluded studies involving pediatric patients, pre-dialysis CKD patients, acute kidney injury patients, and ESRD patients who had received a transplant.

### 2.2. Search Strategy

A medical librarian conducted comprehensive research to identify studies that compared all-cause mortality in patients on maintenance hemodialysis with or without frailty. Research was conducted in April 2024 using the following databases: MEDLINE via PubMed (1985 to present) and Web of Science Core Collection via Clarivate (1985 to present) and a combination of keywords to capture hemodialysis, frailty, and mortality (Table S1).

### 2.3. Data Extraction

Database screening and exclusion of duplicated results were performed by a qualified medical librarian. Two investigators screened the initial search results for data inclusion and performed data extraction independently. Disagreements were resolved by a third author, who also checked the extracted data for accuracy. The full texts of the selected studies were retrieved for a second round of eligibility screening. Reference lists of articles were also searched to identify other relevant studies.

### 2.4. Outcomes

We defined the following outcomes:

1. Comparison of all-cause mortality among patients on maintenance hemodialysis with and without frailty;
2. Comparison of all-cause hospitalization among patients on maintenance hemodialysis with and without frailty.

### 2.5. Quality Assessment and Risk of Bias

The quality of reporting for each study was analyzed by two researchers using the Newcastle-Ottawa Quality Assessment Scale to assess the risk of bias in observational studies (Table S2). Funnel plots were generated to assess publication bias in the included studies.

### 2.6. Statistical Analysis

Statistical analysis was performed using the Statistical Package for Social Science (SPSS 22.0; SPSS Inc., Chicago, IL, USA) and Microsoft Excel (Version 16.45). Statistical Analysis Mortality data were combined as odds ratios (ORs). If ORs were not reported in a study, we calculated ORs from raw mortality or other specific events, if such data was available. If raw data was not available, then ORs were calculated from the provided risk ratio (RR) or hazard ratio (HR) values based on previously published methods [30,31]; this involved converting HRs over different periods into the number of events at 12 months in order to calculate Ors using the relationship between HRs and ORs. Statistical heterogeneity among studies was assessed with Cochran’s *Q* and quantified with the Higgins *I*^2^ statistic. Two different Forest/Funnel plots were shown, with the first including only studies reporting HRs after 1 year of observation and the second including all studies after the conversion of HRs over a long period to ORs at 1 year. ORs were combined using inverse variance with both random and fixed effects models (because the moderate heterogeneity *I*^2^ = 64%, only the figures of random effect models were reported). Publication bias was assessed graphically using Funnel plots and qualitatively using Egger’s regression and the Begg rank correlation method.

## 3. Results

### 3.1. Literature Search

**Figure 1:**
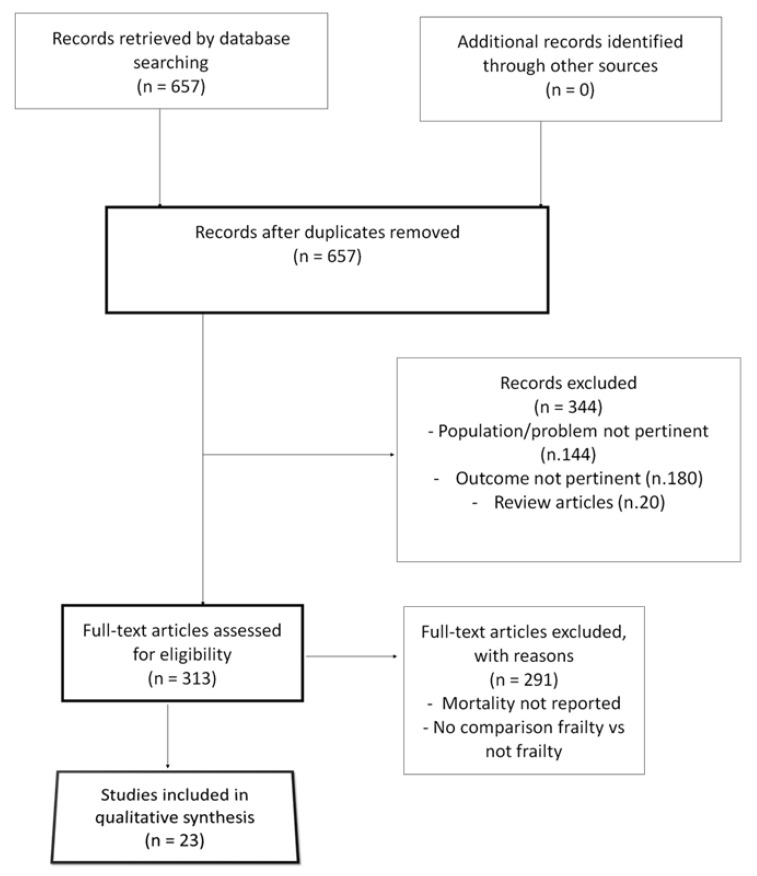
Preferred Reporting Items for Systematic Reviews and Meta-Analyses (PRISMA) flowchart of our analysis.

We screened the titles and abstracts of 657 studies, and 344 were deemed irrelevant (Figure 1). A full-text assessment was performed on 313 studies, and 291 of these were excluded. We included 23 studies in the analysis, with a total of 10,333 patients (5592 frail and 4741 non-frail) [8,9,10,11,12,13,14,15,16,17,18,19,20,21,22,23,24,25,26,27,28,29].

### 3.2. Characteristics of the Studies Included

All data on mortality by frailty were from cohort studies. Eight studies were conducted in Asia, seven in North America, six in Europe, and one in South America. All studies were published after 2011. Frailty was assessed through various tools (Table S3). The Fried Frailty Phenotype was used in 16 studies, the Clinical Frailty Scale in 2 studies, the Frailty Index in 1 study, the Edmonton Frail Scale in 1 study, the Frail Scale in 1 study, and Multiple Frailty Domains in 1 study (Table 1). The length of follow-up was ≤10 months in one study, between 11 and 15 months in seven studies, between 16 and 20 months in three studies, between 21 and 25 months in four studies, between 26 and 30 months in one study, between 31 and 40 months in four studies, and >41 months in two studies. The prevalence of frailty ranged from 17.8% to 90.2%. When frailty was assessed through the Fried Frailty Phenotype, the prevalence of frailty ranged from 24.1% to 90.2%. All studies were rated as having a low risk of bias (Table 2).

All data on hospitalization were from cohort studies. Six studies were conducted in Asia, three in North America, four in Europe, and two in South America. All studies were published after 2011 (Table 1).

Frailty was assessed through various tools (Table S3). The Fried Frailty Phenotype was used in nine studies, the Clinical Frailty Scale in one study, the Frailty Index in one study, the Edmonton Frail Scale in one study, the Frail Scale in two studies, and Multiple Frailty Domains in one study (Table 1). The length of follow-up was ≤10 months in two studies, between 11 and 15 months in five studies, between 16 and 20 months in one study, between 21 and 25 months in three studies, and between 31 and 40 months in four studies. The prevalence of frailty ranged from 25.2% to 88.1%. All studies were rated as having a low risk of bias (Table 2).

### 3.3. Frailty and All-Cause Mortality

**Figure 2:**
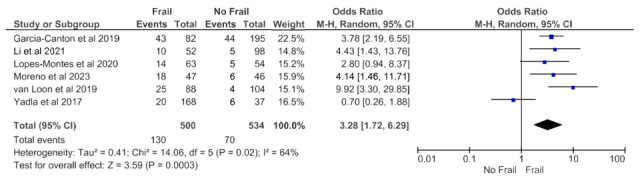
Forest plot of adjusted mortality in frail and non-frail patients on chronic hemodialysis. Studies with a 12-month follow-up period.

Adjusted mortality data that accounted for patient characteristics and treatment variables was available from six studies (1034 patients) with a follow-up period of ≤12 months and revealed an increased all-cause mortality risk in frail patients in the random effects model (pooled OR, 3.28; 95% CI, 1.72–6.29) (Figure 2). Moderate heterogeneity was observed in this analysis (Chi^2^ = 14.06, df = 5; *p* = 0.02; I^2^ = 64%).

**Figure 3:**
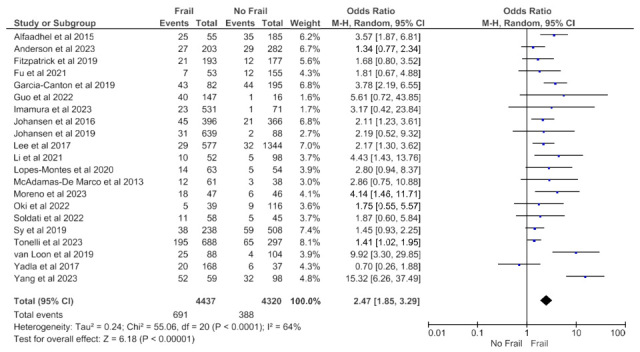
Forest plot of adjusted mortality in frail and non-frail patients on chronic hemodialysis. Studies with any follow-up period.

Adjusted mortality data that accounted for patient characteristics and treatment variables was available from 21 studies (8757 patients) with any follow-up period and revealed an increased all-cause mortality risk in frail patients in the random effects model (pooled adjusted OR 2.47; 95% CI, 1.85–3.29; *p* < 0.00001) (Figure 3).

**Figure 4:**
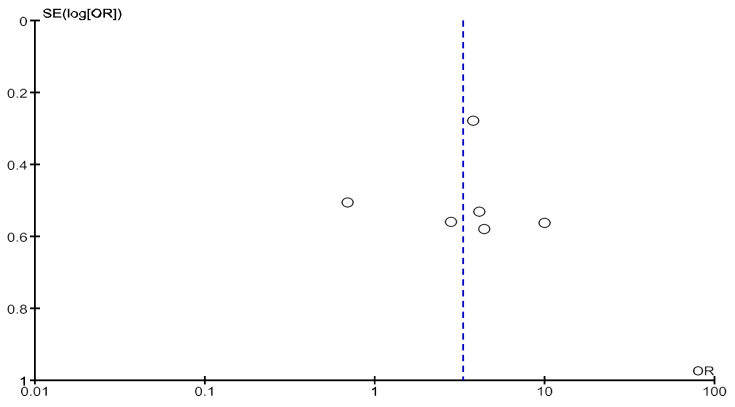
Funnel plot of studies with a follow-up period of 12 months included in the analysis of adjusted mortality.

**Figure 5:**
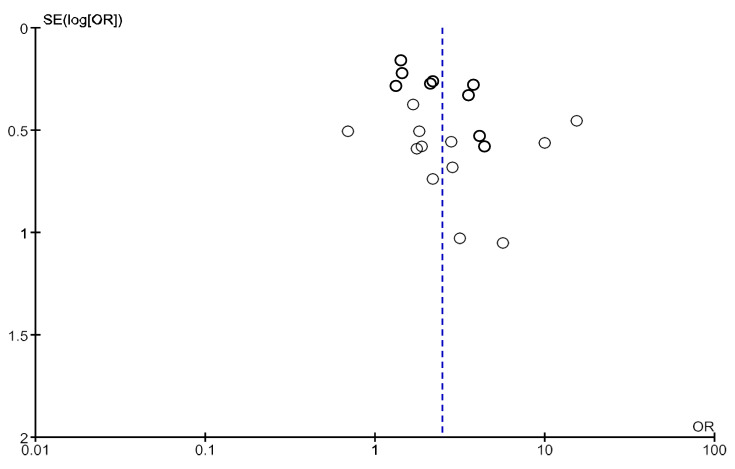
Funnel plot of studies with any follow-up period included in the analysis of adjusted mortality.

High heterogeneity was observed in this analysis (Chi^2^ = 55.1, df = 20; *p* < 0.0001; I^2^ = 64%). As shown in Figure 4 and Figure 5, no obvious asymmetry, indicating no clear evidence of publication bias, was observed.

**Figure 6:**
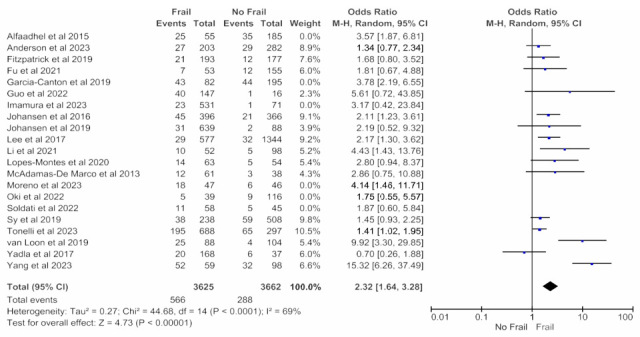
Forest plot of adjusted mortality in frail and non-frail patients on chronic hemodialysis. Studies with any follow-up period. Frailty assessed with Fried Frailty Phenotype.

**Figure 7:**
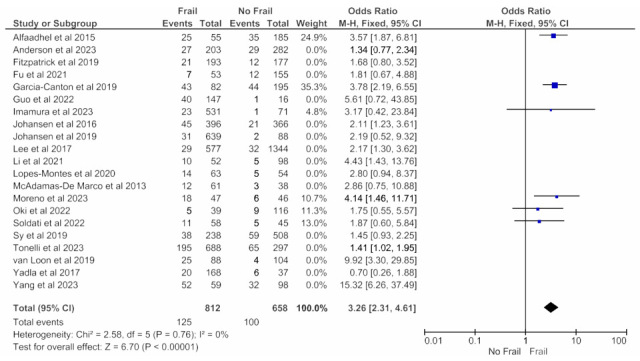
Forest plot of adjusted mortality in frail and non-frail patients on chronic hemodialysis. Studies with any follow-up period. Frailty assessed with tools other than Fried Frailty Phenotype.

Figure 6 and Figure 7 show, respectively, the meta-analyses on the association between frailty and mortality when frailty was assessed with the Fried Frailty Phenotype (Figure 6) or with other tools (Figure 7). Both analyses revealed an increased all-cause mortality risk in frail patients.

### 3.4. Frailty and All-Cause Hospitalization

**Figure 8:**
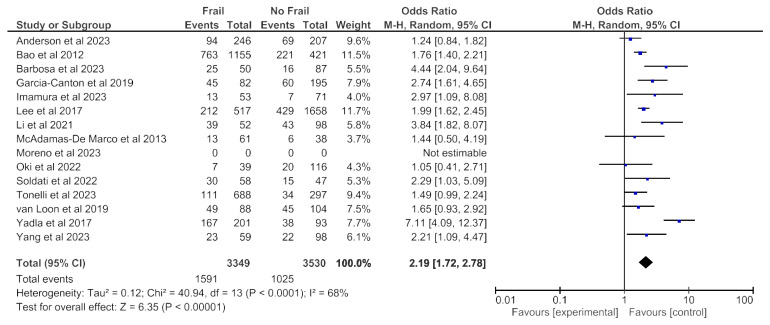
Forest plot of hospitalization in frail and non-frail patients on chronic hemodialysis. Studies with a 12-month follow-up period.

**Figure 9:**
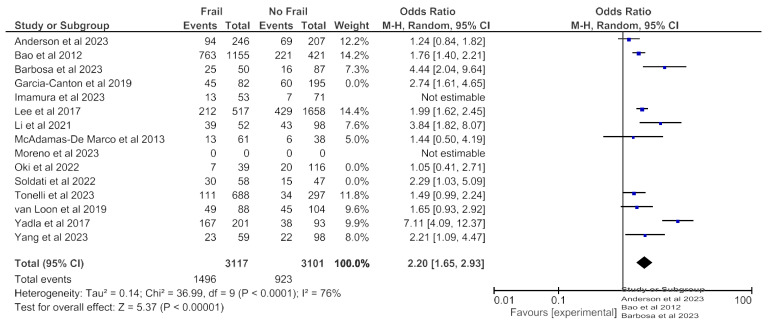
Forest plot of hospitalization in frail and non-frail patients on chronic hemodialysis. Frailty assessed with Fried Frailty Phenotype.

**Figure 10:**
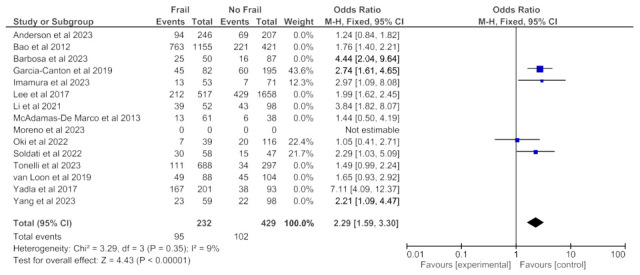
Forest plot of hospitalization in frail and non-frail patients on chronic hemodialysis. Frailty assessed with tools other than Fried Frailty Phenotype.

All-cause hospitalization data was available from 15 studies (6349 patients) with any follow-up period and revealed an increased all-cause hospitalization risk in frail patients in the random effects model (pooled adjusted OR 2.19; 95% CI, 1.72–2.78) (Figure 8). Moderate heterogeneity was observed in this analysis (Chi^2^ = 40.9, df = 13; *p* < 0.0001; I^2^ = 68%). Figure 9 and Figure 10 show, respectively, the meta-analyses on the association between frailty and hospitalization when frailty was assessed with the Fried Frailty Phenotype (Figure 9) or with other tools (Figure 10). Both analyses revealed an increased all-cause hospitalization risk in frail patients.

**Figure 11:**
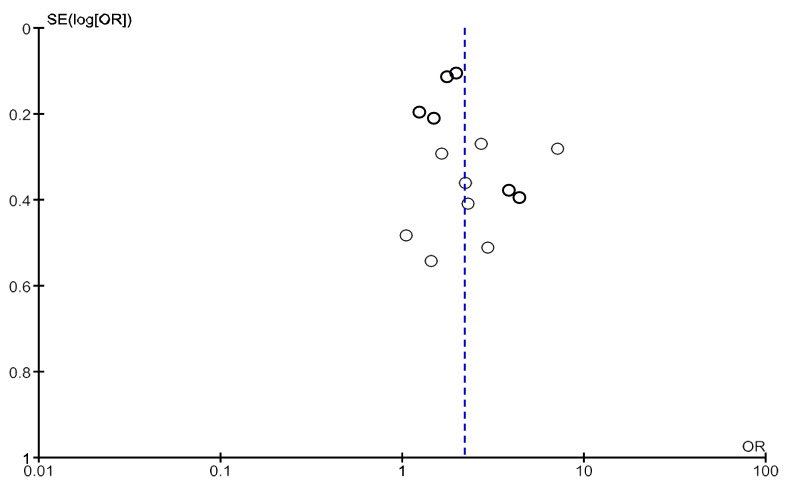
Funnel plot of studies with a follow-up period of 12 months included in the analysis of hospitalization.

High heterogeneity was observed in the analysis including only one study in which frailty was assessed through the Fried Frailty Phenotype (Chi^2^ = 44.6, df = 14; *p* < 0.0001; I^2^ = 69%). No heterogeneity was observed in the analysis including only study in which frailty was assessed through other tools (Chi^2^ = 2.58, df = 5; *p* = 0.76; I^2^ = 0%). As shown in Figure 11, no obvious asymmetry, indicating no clear evidence of publication bias, was observed.

## 4. Discussion

The present systematic review shows that frailty is associated with an increased risk of all-cause mortality in patients on chronic hemodialysis. These results agree with those of the meta-analysis by Lee et al. published in 2021, which included seven studies and reported the hazard ratio and not the odds ratio [5]. Similarly, the recent meta-analysis by Cheng et al., which included three studies and reported the odds ratio, showed that frailty was associated with a 2.33-fold higher risk of mortality [6]. The systematic review by He et al. included both patients receiving hemodialysis and peritoneal dialysis, thus making it difficult to compare the results [7]. In the present study, we showed two different Forest/Funnel plots, with the first including only studies reporting HRs after 1 year of observation and the second including all studies after the conversion of HRs over a long period to ORs at 1 year.

The present study also shows that frailty is associated with an increased risk of all-cause hospitalization. Hospitalizations were often due to falls, fracture, cardiovascular events, infectious events, and vascular access complications [32] and were associated with a deterioration in the quality of life of both patients and siblings and a significant increase in health care cost expenditures [33]. The association between frailty and hospitalization has been primarily demonstrated in chronic diseases such as heart failure, chronic obstructive pulmonary disease, hypertension, liver failure, and inflammatory bowel diseases [34,35,36,37,38].

The observation that frailty is associated with both all-cause mortality and hospital raises significant concerns, given that frailty is common in patients on maintenance hemodialysis: the prevalence ranged between 22.9% and 90.2% in the studies included in the present review [8,9,10,11,12,13,14,15,16,17,18,19,20,21,22,23,24,25,26,27,28,29].

Numerous factors contribute to the onset or the progression of frailty in the general population and in patients on maintenance hemodialysis, such as female sex, low education, low socioeconomic position, living alone, loneliness, multimorbidity, malnutrition, obesity, impaired cognition, depressive symptoms, physical inactivity, smoking, increased alcohol intake, inflammation, and micronutrients deficits [1,2]; these factors likely help explain why frailty increases the risk of mortality in these populations. However, other mechanisms closely related to age may also explain the multidimensional nature of frailty and the associated increased risk of mortality [1,2].

The results of the present systematic review raise two important questions concerning routine clinical practice. First, should we assess frailty in all patients starting maintenance hemodialysis? Although it is evident that knowing whether a patient is frail or not gives physicians the opportunity to better define the prognosis, frailty remains to be defined, especially in patients on chronic hemodialysis; it is also unclear whether such an approach will modify the outcomes. Second, should we try to treat frailty through pharmacological and non-pharmacological therapeutic strategies? Most of the factors contributing to frailty can be treated or prevented [1,2]. There is evidence from some studies that treating depressive symptoms, improving micronutrient intake, and addressing malnutrition/physical inactivity in patients on chronic hemodialysis can yield positive results [39,40,41]. However, the certainty of evidence regarding the effects on muscle strength, falls, disability, gait speed, and frailty in general is low or very low in patients on maintenance hemodialysis [39,40,41]. Overall, it seems that the high frequency of frailty in patients on maintenance hemodialysis, the high associated costs, and the high risk of associated adverse outcomes indicate the need for clinical trials to identify effective treatments.

Interestingly, a significant association between frailty and mortality was demonstrated both when considering all studies together and when analyzing studies in which frailty was assessed by the Fried Frailty Phenotype or other tools separately (Figure 7 and Figure 8). These results clarify that the observed associations are consistent regardless of the method used and may inform clinical decision making regarding the choice of frailty assessment tools in nephrology practice.

The present review has some limitations. First, the number of studies included in the review was relatively small. Second, we only assessed the risk of all-cause mortality and not cardiovascular-related mortality or infectious disease-related mortality due to the limited number of studies that assessed such outcomes. Third, the heterogeneity was moderate to high in some meta-analyses but not in all. This may depend on several factors such as the length of the follow-up period, the characteristics of the study populations, and the tools used to assess frailty.

In conclusion, the present systematic review and meta-analysis show that frailty is a significant risk factor for all-cause mortality and all-cause hospitalization in patients on maintenance hemodialysis and presents clinicians with important challenges in routine clinical practice.

## Figures and Tables

**Table 1:** Characteristics of the studies on frailty and all-cause mortality and all-cause hospitalization. * Data are expressed as mean ± SD or median [95% CI]. HD, hemodialysis; PD, peritoneal dialysis; M, mortality; H, hospitalization.

	Country	Type of Study	Type of Dialysis	Number of Patients	Age (yrs) *	Sex (% of Males)	Frailty Assessment	Prevalence of Frailty (%)	Outcome	Duration of Follow-Up (Months)
Bao, 2012	USA	Cohort	HD	1576	59.6 ± 14.2	55.5	Fried Frailty Phenotype	73	M, H	33
McAdams-De Marco, 2013	USA	Cohort	HD	99	60.6 ± 13.6	53.4	Fried Frailty Phenotype	61.6	M, H	36
Alfaadhel, 2015	Canada	Cohort	HD	240	62 ± 14	65.4	Clinical Frailty Scale	22.9	M	19
Johansen, 2016	USA	Cohort	HD	762	57.1 ± 14.1	59.3	Fried Frailty Phenotype	51.2	M	19
Yadla, 2017	India	Cohort	HD	205	44.9 ± 13.2	69.2	Fried Frailty Phenotype	81.9	M, H	12
Lee, 2017	Korea	Cohort	HD PD	1658 (403 on PD)	55.2 ± 11.9	55.7	Fried Frailty Phenotype	64.1	M, H	17
Garcia-Canton, 2019	Spain	Cohort	HD	224	65 [15.6–74.5]	65.3	Edmonton Frail Scale	36.6	M, H	12
Johansen, 2019	USA	Cohort	HD	727	57.2 ± 14.3	54.8	Fried Frailty Phenotype	87.9	M	75
Fritzpatick, 2010	USA	Cohort	HD	370	54.9 ± 13.1	58.4	Fried Frailty Phenotype	52.2	M	30
Van Loon, 2019	The Netherlands	Cohort	HD	192	75 ± 7	67	Fried Frailty Phenotype	45.8	M, H	12
Lopez-Montes, 2020	Spain	Cohort	HD	117	78.1 ± 4.1	63.2	Fried Frailty Phenotype	53.8	M	12
Sy, 2019	USA	Cohort	HD	746	57.2 ± 14.2	59.4	Fried Frailty Phenotype	31.9	M	74
Fu, 2021	China	Cohort	HD	103	60.5 ± 12.7	54.3	Fried Frailty Phenotype	46.9	M	25
Li, 2021	China	Cohort	HD	150	69 [64–75]	48	Fried Frailty Phenotype	34.6	M, H	12
Soldati, 2022	Italy	Cohort	HD	103	79.1 ± 7.6	45	Frailty Index	56.3	M, H	21
Guo, 2022	China	Cohort	HD	163	71.6 ± 5.9	55.4	Fried Frailty Phenotype	90.2	M	13
Oki, 2022	Japan	Cohort	HD	155	66.7 ± 14.1	71	Clinical Frailty Scale	25.2	M, H	24
Tonelli, 2023	Canada	Cohort	HD	985	62	61.2	Fried Frailty Phenotype	69.8	M, H	33
Anderson, 2023	UK	Cohort	HD	448	62	57.4	Fried Frailty Phenotype	42.1	M, H	22
Imamura, 2023	Japan	Cohort	HD	603	72.4 ± 7.8	61	Multiple frailty domains	88.1	M, H	37
Moreno, 2023	Colombia	Cohort	HD	93	64 [53–69]	59.1	Frail Scale	50.5	M, H	12
Yang, 2023	China	Cohort	HD	157	67 [63–74]	51	Fried Frailty Phenotype	37.6	M, H	3
Barbosa, 2023	Brazil	Cohort	HD	137	61		Frail scale	58.4	M, H	9

**Table 2:** Newcastle–Ottawa quality assessment of individual studies. It evaluates the risk of bias across three main domains: selection of study groups, comparability of groups, and ascertainment of outcomes (or exposures). The NOS assigns a score (ranging from 0 to 9 stars), with higher scores generally indicating higher quality.

	Selection	Selection	Selection	Selection	Comparability	Comparability	Outcome	Outcome	Outcome	
Study	Representativeness of the Exposed Cohort	Selection of the Non-Exposed Cohort	Ascertainment of Exposure	Outcome of Interest not Present at Start	Study Controls for Level of Acute Illness	Study Controls for Any Additional Factor	Assessment of Outcome	Follow-Up Long Enough for Outcomes to Occur	Adequacy of Follow Up of Cohorts	Risk of Bias
McAdams-De Marco, 2013	a	a	a	b	a	a	a	a	a	low
Alfaadhel, 2015	a	a	a	b	a	a	a	a	a	low
Johansen, 2016	a	a	a	b	a	a	a	a	a	low
Yadla, 2017	a	a	a	b	a	a	a	a	a	low
Lee, 2017	a	a	a	b	a	a	a	a	a	low
Garcia-Canton, 2019	a	a	a	b	a	a	a	a	a	low
Johansen, 2019	a	a	a	b	a	a	a	a	a	low
Fritzpatick, 2010	a	a	a	b	a	a	a	a	a	low
Bao, 2012	a	a	a	b	a	a	a	a	a	low
Van Loon, 2019	a	a	a	b	a	a	a	a	a	low
Lopez-Montes, 2020	a	a	a	b	a	a	a	a	a	low
Sy, 2019	a	a	a	b	a	a	a	a	a	low
Fu, 2021	a	a	a	b	a	a	a	a	a	low
Li, 2021	a	a	a	b	a	a	a	a	a	low
Soldati, 2022	a	a	a	b	a	a	a	a	a	low
Guo, 2022	a	a	a	b	a	a	a	a	a	low
Oki, 2022	a	a	a	b	a	a	a	a	a	low
Tonelli, 2023	a	a	a	b	a	a	a	a	a	low
Anderson, 2023	a	a	a	b	a	a	a	a	a	low
Imamura, 2023	a	a	a	b	a	a	a	a	a	low
Moreno, 2023	a	a	a	b	a	a	a	a	a	low
Yang, 2023	a	a	a	b	a	a	a	a	a	low

## Data Availability

The datasets generated during and/or analyzed during the current study are available from the corresponding author on reasonable request.

## Supplementary Materials

The following supporting information can be downloaded at: https://www.mdpi.com/article/10.3390/jcm14144914/s1, Table S1: Search strategy.; Table S2: Risk of bias was assessed with the Newcastle–Ottawa Assessment Scale using the questions above. The procedure for converting the responses to an overall risk of bias assessment (i.e., low, medium, or high risk of bias) is detailed as well.; Table S3: Tools to assess the presence of frailty.
