# Supplementary material for: Frailty, All-Cause Mortality, and Hospitalization in Patients on Maintenance Hemodialysis: A Systematic Review and Meta-Analysis
Source: J Clin Med. 2025 Jul 10;14(14):4914. doi: 10.3390/jcm14144914 (PMC12294973; doi:10.3390/jcm14144914)
Supplement: Supplementary file 1 [file jcm-14-04914-s001.zip › jcm-3713627-supplementary.pdf]

Database: MEDLINE (via PubMed)

Set #	Search Strategy	Results
**#1**<br><br>**hemodialysis**	hemodialysis OR haemodialysis	188564
**#2**<br><br>**frailty**	frailty OR frail	36246
**#3**<br><br>**mortality**	Mortality OR mortalities OR fatal OR fatality OR fatalities OR death OR deaths	2649490
**#4**<br><br>**hospitalization**	hospitalization	7807447
**#5**	#1 AND #2	657
**#6**	#1 AND #2 AND #3	338
**#7**	#1 AND #2 AND #4	497

Database: Web of Science Core Collection

Set #	Search Strategy	Results
**#1**<br><br>**hemodialysis**	hemodialysis	115767
**#2**<br><br>**frailty**	frailty	44552
**#3**<br><br>**mortality**	mortality	1449942
**#4**<br><br>**hospitalization**	hospitalization	259477
**#5**	#1 AND #2	752

**#6**	#1 AND #2 AND #3	408
**#7**	1 AND #2 AND #4	133

Table S1. Search strategy.

Selection

1. Representativeness of the exposed cohort a. Truly representative of the average patient in the community (*) b. Somewhat representative of the average patient in the community (*) c. Selected group of patients d. No description of the derivation of the cohort
2. Selection of the non-exposed cohort a. Drawn from the same community as the exposed cohort (*) b. Drawn from a different source c. No description of the derivation of the non-exposed cohort 3. Ascertainment of exposure a. Secure record (e.g. medical records) (*) b. Structured interview (*) c. Written self-report d. No description
4. Demonstration that outcome of interest was not present at start of study a. Yes (*) b. No

Comparability of cohorts on basis of design or analysis
1. Study controls for level of acute illness a. Yes (*) b. No
2. Study controls for any additional factor. a. Yes (*) b. No

Outcome
1. Assessment of outcome a. Independent blind assessment (*) b. Record linkage (*) c. Self-report d. No description
2. Was follow-up long enough for outcomes to occur a. Yes (*) b. No
3. Adequacy of follow up of cohorts a. Complete follow up (all subjects accounted for) (*) b. Subjects lost to follow up unlikely to introduce bias (≤10% lost to follow-up, or description provided of those lost) (*) c. Follow up rate < 90% and no description of those lost d. No statement

Thresholds used to convert the Newcastle–Ottawa scale to categories (good, fair, and poor):
Good quality/low risk of bias: 3 or 4 stars in selection domain AND 1 or 2 stars in comparability domain AND 2 or 3 stars in outcome/exposure domain.
Fair quality/medium risk of bias: 2 stars in selection domain AND 1 or 2 stars in comparability domain AND 2 or 3 stars in outcome/exposure domain.
Poor quality/high risk of bias: 0 or 1 star in selection domain OR 0 stars in comparability domain OR 0 or 1 star in outcome/exposure domain.

Table S2. Risk of bias was assessed with the Newcastle–Ottawa Assessment Scale using the questions above. The procedure for converting the responses to an overall risk of bias assessment (i.e., low, medium, or high risk of bias) is detailed as well.

Frailty Phenotype	Five items: weight loss, low physical activity, exhaustion, slowness, weakness	Frailty: ≥3 items; pre-frailty: 1–2 items; robust: 0 items
**Frailty Index**	**30 or more accumulated health deficits**	**Number of health deficits present ÷ number of health deficits measured**<br><br>**Score ranges from 0 (no deficits) to 1 (all deficits)**<br><br>**Suggested cutoff score for frailty >0.25**
**Clinical Frailty Scale**	**1 Very Fit**<br><br>**People who are robust, active, energetic and motivated. These people commonly exercise regularly. They are among the fittest for their age.**<br><br>**2 Well**<br><br>**People who have no active disease symptoms but are less fit than category 1. Often, they exercise or are very active occasionally, e.g. seasonally.**<br><br>**3 Managing Well**<br><br>**People whose medical problems are well controlled, but are not regularly active beyond routine walking.**<br><br>**4 Vulnerable**<br><br>**While not dependent on others for daily help, often symptoms limit activities. A common complaint is being "slowed up", and/or being tired during the day.**<br><br>**5 Mildly Frail**<br><br>**These people often have more evident slowing, and need help in high order IADLs (finances, transportation, heavy housework, medications). Typically, mild frailty progressively impairs**	**Frailty: score ≥5**

	**shopping and walking outside alone, meal preparation and housework.**<br><br>**6 Moderately Frail**<br><br>**People need help with all outside activities and with keeping house. Inside, they often have problems with stairs and need help with bathing and might need minimal assistance (cuing, standby) with dressing.**<br><br>**7 Severely Frail**<br><br>**Completely dependent for personal care, from whatever cause (physical or cognitive). Even so, they seem stable and not at high risk of dying (within ~ 6 months).**<br><br>**8 Very Severely Frail**<br><br>**Completely dependent, approaching the end of life. Typically, they could not recover even from a minor illness.**<br><br>**9. Terminally Ill**<br><br>**Approaching the end of life. This category applies to people with a life expectancy**	
**Edmonton Frailty Scale**	**Nine items: cognition, general health status (number of hospitalizations), functional independence, social support, medication use, nutrition, mood, continence, functional performance**	**Frailty: score ≥7**

Table S3. Tools to assess the presence of frailty.
